# Development and validation of an index of musculoskeletal functional limitations

**DOI:** 10.1186/1471-2474-10-62

**Published:** 2009-06-06

**Authors:** Jeffrey N Katz, Elizabeth A Wright, John A Baron, Elena Losina

**Affiliations:** 1 Department of Orthopaedic Surgery and Division of Rheumatology, Brigham and Womens' Hospital, and Department of Epidemiology, Harvard School of Public Health, Boston, USA; 2 Department of Orthopaedic Surgery and Division of Rheumatology, Brigham and Womens' Hospital, Boston, USA; 3 Department of Community and Family Medicine, Dartmouth Medical School, Lebanon, USA; 4 Department of Orthopaedic Surgery and Division of Rheumatology, Brigham and Womens' Hospital and Department of Biostatistics, Boston University School of Public Health, Boston, USA

## Abstract

**Background:**

While musculoskeletal problems are leading sources of disability, there has been little research on measuring the number of functionally limiting musculoskeletal problems for use as predictor of outcome in studies of chronic disease. This paper reports on the development and preliminary validation of a self administered musculoskeletal functional limitations index.

**Methods:**

We developed a summary musculoskeletal functional limitations index based upon a six-item self administered questionnaire in which subjects indicate whether they are limited a lot, a little or not at all because of problems in six anatomic regions (knees, hips, ankles and feet, back, neck, upper extremities). Responses are summed into an index score. The index was completed by a sample of total knee replacement recipients from four US states. Our analyses examined convergent validity at the item and at the index level as well as discriminant validity and the independence of the index from other correlates of quality of life.

**Results:**

782 subjects completed all items of the musculoskeletal functional limitations index and were included in the analyses. The mean age of the sample was 75 years and 64% were female. The index demonstrated anticipated associations with self-reported quality of life, activities of daily living, WOMAC functional status score, use of walking support, frequency of usual exercise, frequency of falls and dependence upon another person for assistance with chores. The index was strongly and independently associated with self-reported overall health.

**Conclusion:**

The self-reported musculoskeletal functional limitations index appears to be a valid measure of musculoskeletal functional limitations, in the aspects of validity assessed in this study. It is useful for outcome studies following TKR and shows promise as a covariate in studies of chronic disease outcomes.

## Background

Observational studies of the effects of interventions must take into account a wide range of factors that can also influence health outcomes in chronic disease. These include specific medical comorbidities, such as cardiovascular, respiratory, endocrine and other disorders; mental health, including anxiety and depression; and socioeconomic factors such as educational attainment, income and health insurance status. A large body of research has established methods for the measuring and adjusting for these domains [1-9].

Musculoskeletal problems have received less attention as potential correlates of a wide range of health outcomes in subjects with chronic diseases. We define musculoskeletal limitations as functionally limiting problems involving bones, joints, cartilage, soft tissues and other musculoskeletal structures. Examples include various forms of arthritis, tendonitis, bursitis, fracture, and regional musculoskeletal pain (low back, neck, shoulder, foot, knee, etc). While musculoskeletal problems are among the leading sources of disability, particularly in the elderly [10-21], there has been little work to date on measuring the number of limiting musculoskeletal problems and using such measures as covariates in studies of chronic disease outcomes. Several scales measure functional status, a key outcome in patients with musculoskeletal conditions. But these scales do not attempt to capture the number of limiting musculoskeletal problems per se [22-24]. A single summary musculoskeletal limitations index score would provide an analytically efficient approach to accounting for this important domain. The index score could serve as a covariate to reflect the effect of musculoskeletal problems and attendant limitations on salient health outcomes.

The objective of this paper is to report on the development and preliminary validation of a self-report index of musculoskeletal functional limitations that aggregates the patient's functionally limiting musculoskeletal problems into a single score. We developed this index in the context of survey research on the outcome of total knee replacement in a cohort of US Medicare beneficiaries who had undergone elective total knee replacement two years prior to their participation in the survey.

## Methods

### Development of the index of musculoskeletal limitation

The index was developed by a group of orthopedic surgeons, rheumatologists, physical therapists, survey research personnel and methodologists. We conceptualized musculoskeletal functional limitation as the aggregation of individual regional musculoskeletal problems that influence subjects' ability to perform daily activities. Our approach to measuring this domain was to ask patients whether problems in specific anatomic regions (e.g. hip, back, neck) limited their daily activities. Subjects responded whether they were not limited, limited a little or limited a lot by problems in that anatomic area. Since this particular population had undergone total knee replacement, we listed both right and left knee and considered only the knee contralateral to the replaced one in the musculoskeletal limitations index. The structure of the questionnaire is shown in Table 1. The questionnaire is scored by summing the responses to each item (not limited = 0 points; limited a little = 1 point; limited a lot = 2 points) across all six items. The index has a theoretical range in score from 0 to 12. We pilot tested the instrument for acceptability in subjects with musculoskeletal problems.

**Table 1:** Subjects responses* to the questions on the musculoskeletal functional limitations index.

Area	Has not limited my activities	Limited my activities a little	Limited my activities a lot	Missing
Knee**	326 (35%)	341 (37%)	231 (25%)	34 (3.7%)

Hips	549 (59%)	222 (24%)	88 (9.4%)	73 (7.8%)

Back	434 (47%)	267 (29%)	157 (17%)	74 (7.9%)

Hands, wrists, arms or shoulders	508 (55%)	269 (29%)	73 (7.8%)	82 (8.8%)

Feet and ankles	535 (57%)	229 (25%)	92 (9.9%)	76 (8.2%)

Neck	649 (70%)	159 (17%)	44 (4.7%)	80 (8.6%)

* Subjects are asked: "In the past four weeks, how much have problems in any of the following areas limited your activities?" (responses shown for all 932 respondents)

**contralateral to the operated knee

### Patients

The source population for the study included Medicare recipients who had primary TKR in Ohio, Illinois, North Carolina or Tennessee in calendar year 2000. We used Medicare claims submitted by hospitals (Medicare Part A) or by surgeons (Medicare Part B) in the year 2000 to identify patients having primary TKR, as reported previously [25]. We excluded patients with claims indicating pre-existing infection of the knee, metastatic cancer or bone cancer. To obtain complete claims histories, we also excluded patients who were enrolled in health maintenance organizations (HMOs), not enrolled in both parts of Medicare, under 65 or not resident in the United States. We also excluded patients having bilateral primary TKRs performed in the same hospitalization.

We stratified hospitals according to annual hospital volume of total knee replacement in the Medicare population. We randomly selected hospitals from these volume strata with probability proportional to number of discharges for TKR. We then randomly selected patients from hospitals to yield a stratified random sample.

### Data Sources and Data Elements

#### Medicare claims

Claims data provided information on patient age, sex, arthritis diagnosis, medical comorbidity (assessed with a claims-based version of the Charlson comorbidity index [1,26] and whether the state Medicaid program paid the Medicare premiums. (This indicator identifies patients with income near the poverty level.)

#### Survey

In 2002, two years after the subjects' procedures, the research team sent a letter inviting patients to participate, along with a book of ten postage stamps as a response incentive. Patients were asked to return the letter and indicate whether they wished to participate. Per protocol established by the Center for Medicare and Medicaid Services, the research team was not permitted to phone patients who either refused or never responded to the letters of invitation. The team sent three letters of invitation and included a survey questionnaire in the third.

#### Survey questionnaire data

The questionnaire covered a wide range of topics. Most relevant to these analyses, the questionnaire included the musculoskeletal functional limitations index, as described above. It also assessed lower extremity functional status and pain with the Functional Status and Pain Scales of the WOMAC (Western Ontario and McMaster Universities Osteoarthritis Index) [22,27], a widely used measure of lower extremity pain and functional status in patients with osteoarthritis. The questionnaire included the Basic Activities of Daily Living Scale and the Intermediate Activities of Daily Living Scale from the Functional Status Questionnaire [23]. The Basic ADL Scale has three items that ask about eating, dressing or bathing; transferring from bed or chair; and walking indoors. The Intermediate ADL Scale has six items that ask about walking several blocks; walking one block or several flights of stairs; doing work around the house; doing errands; driving a care or using public transportation; and doing vigorous activities. For each of these functional status scales, we took the mean response score of all of the items in the scale and transformed scores to 0–100, with 100 representing the best possible score. We asked patients to report on their overall health using a 0 to 100 scale, where 100 represented "the best health you can imagine" and 0 represented "the worst health you can imagine (death)." The questionnaire contained the five item mental health subscale of the SF-36, a valid measure of symptoms of depression and anxiety [28]. The questionnaire also contained a self report multi-item geriatric functional index that we modeled on prior work [29]. It queried patients about typical geriatric functional problems including incontinence and limitation due to fear of falling, poor vision, poor hearing, muscle weakness and medical conditions.

The questionnaire also had an item asking respondents how often they participated in recreational aerobic exercise for at least a half hour at a time, with aerobic exercise including walking, swimming, dancing, biking or other sports. The possible responses were never, less than once a week, a few times week and almost every day. We also asked patients whether in the last four weeks they used any musculoskeletal supportive devices for ambulation (such as a cane; yes or no). We asked how often they fell all the way to the ground or fell and hit a chair or stair in the last year (never, once, 2–3 times, > 3 times). Finally we asked patients how often they depended upon another person to do household chores, shopping or running errands that they could not do themselves (every day, 2–3 days/week, about 1 day/week, 1–3 times in the past 4 weeks, not at all).

### Analyses

Analyses were performed among subjects that completed all items on the index. We performed sensitivity analyses among subjects that left one or more items from the index missing; findings were essentially unchanged.

#### Missing data

We calculated the number (proportion) of respondents who left each item of the musculoskeletal limitations index missing.

#### Item level validity

We used generalized linear models to examine the association of each item in the musculoskeletal limitations index with the mean score on the Intermediate Activity of Daily Living Scale (IADL). We hypothesized that increasing levels of severity of the ordinally scaled musculoskeletal limitations items would be associated with worsening functional status, as measured by the IADL scale.

#### Index level convergent validity

We examined the correlation of the musculoskeletal functional limitations index score with three measures of functional status, the Basic Activities of Living Scale, the Intermediate Activities of Living Scale, the WOMAC functional status scale and 0–100 scale that captures several reported overall health. These analyses used the Spearman correlation coefficient. We hypothesized that increasingly severe musculoskeletal functional limitation would be associated with worse functional status or overall health, as measured by these instruments.

#### Discriminant validity

We measured the association of the musculoskeletal functional limitations index with self-report level of exercise, use of a walking support, falls and dependence upon others for chores, shopping or errands. We assessed the difference in mean musculoskeletal index scores across the ordinal levels of these variables using generalized linear models. We hypothesized that increasing severity on the musculoskeletal limitations index would be associated with lower frequency of regular exercise, greater use of supportive devices, more frequent falls and more frequent dependence on others.

#### Independence of the association between musculoskeletal comorbidity and other measures of health status

We examined the independent association of the musculoskeletal limitations index with the 0–100 self-reported overall health scale. These analyses adjusted for other measures of health burden, including medical comorbidity (Charlson Index), age and mental health status, as well as knee pain (assessed with the WOMAC pain scale) and sex. We used multiple linear regression with the 0–100 overall health rating scale as the dependent variable and each of the abovementioned variables as independent variables. We hypothesized that the musculoskeletal limitations index would be associated significantly with the overall health scale after adjustment for the other variables in the model.

We also examined the distribution of musculoskeletal functional limitations index scores among subjects with good WOMAC pain and functional status scores (greater than 80 and 70 respectively). We hypothesized that even among these patients with generally good lower extremity pain and functional status, there would be a wide range of musculoskeletal functional limitations scores.

#### Human Studies

This study was approved by the Human Studies Committee of Partners HealthCare and Brigham and Women's Hospital, Boston. Patients received a letter containing the elements of informed consent and returned it to the investigators if there were interested in participating. A formal consent form was not required by the Human Studies Committee because the research was non-interventional and minimal risk.

## Results

### Cohort characteristics

#### Patient Recruitment

As reported previously,[25] 1597 eligible patients were invited to participate. Of these, 230 (14%) refused, 365 (23%) did not respond to three letters of invitation and 1002 (63%) agreed to participate. Of those who agreed, 932 completed surveys (58% of those eligible).

We examined claims data to gain insight into differences between patients who completed surveys and those who either refused to participate or never answered the letters of invitation. Nonresponders tended to be slightly older (mean age 74.8 years) than responders (73.6 years, p = 0.001). Responders were more likely to be white (93%) than non-responders (89%, p = 0.005). Fourteen percent of non-responders received Medicaid supplementation (a surrogate for low income) as compared with just 7% of participants (p < 0.0001).

#### Baseline features

The mean age of the sample was 75 years, (sd 5.5, range 66–94 years). Sixty-four per cent were female. Thirty-one percent of patients used a walking support, 44% engaged in aerobic exercise at least a few times a week, 18% had fallen at least twice in the past year and 24% depended at least once a week upon another person to do chores, shopping or errands. The mean score on the musculoskeletal functional limitations index was 3.1, with median 3, standard deviation 2.7 and range 0 to 12.

#### Missing data on musculoskeletal functional limitations index

Of the 932 patients who completed surveys, 782 (84%) had complete data on all index items. Table 1 shows the responses to each item of the musculoskeletal functional limitations index. The proportion of missing values ranged from 3.7% to 8.8% for each anatomic area. Twenty-six subjects (2.8%) left all items missing.

#### Item level validity

**Figure 1:**
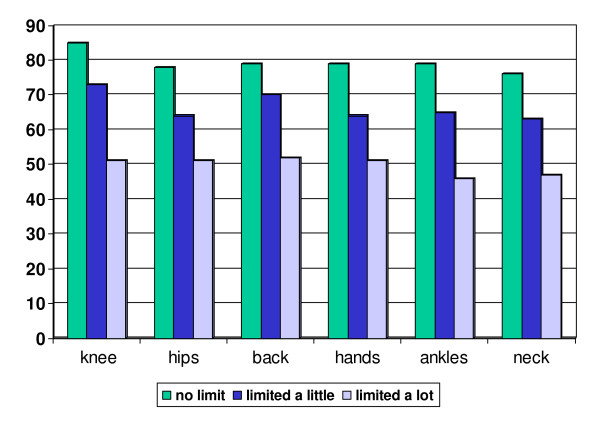
**Mean Intermediate Activity of Daily Living Score stratified by response to each item of the musculoskeletal functional limitations index**.

For each item in the index, the mean Intermediate Activity of Daily Living Score increased monotonically across the levels of severity (0 = no problem; 1 = limits my activities a little; 2 = limits my activity a lot). Each of these associations was highly statistically significant (p < 0.0001 for each). These differences are also highly clinically significant, ranging from an Intermediate ADL score of around 80 for patients who indicated that they did not have the particular musculoskeletal problem to around 50 for patients who indicated that the problem limited their activities a lot (Figure 1).

#### Convergent validity

The musculoskeletal functional limitations index had moderately strong correlations with three other measures of functional status: WOMAC function scale (r = 0.60), Intermediate Activities of Daily Living Scale (r = 0.60) and Basic Activities of Daily Living Scale (r = 0.48) and a moderate correlation with the 0–100 self rated health scale (r = 0.40). Each of these Spearman correlation coefficients was statistically significant (p < 0.0001 for each).

#### Discriminant validity

**Figure 2:**
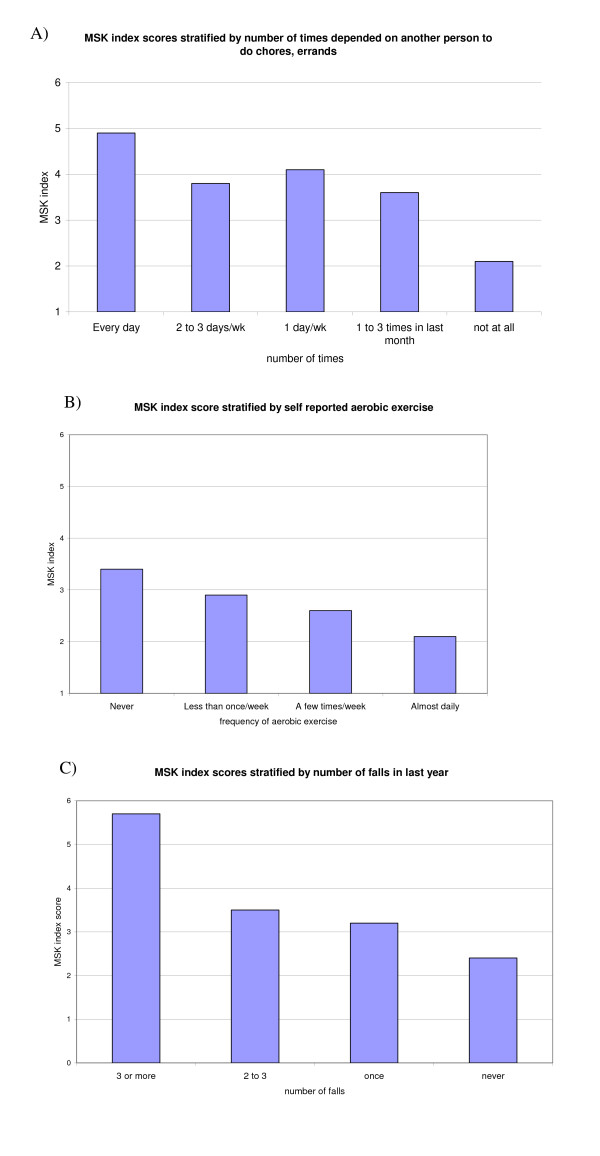
**Mean musculoskeletal functional limitations index score stratified by self reported dependence on others to do chores and errands (2a); self reported aerobic exercise (2b); and self-reported falls in the last year (2c)**.

Subjects that used a walking support had a mean musculoskeletal index score of 4.1 (95% CI 3.8, 4.4), as compared to subjects that did not use a walking support, who had a mean musculoskeletal index score of 2.3 (95% CI 2.1, 2.5). This difference was statistically significant (p < 0.0001). Self-reported exercise was associated with musculoskeletal comorbidity score. Subjects who stated that they never exercise regularly had mean musculoskeletal comorbidity scores of 3.4 (95% CI 3.1, 3.7), whereas subjects who stated that they exercised daily had mean musculoskeletal comorbidity scores of 2.1 (95% CI 1.7, 2.6). Similarly, subjects who reported no falls in the last year had a mean musculoskeletal index score of 2.4 (95% CI 2.2, 2.6) while those who reported more than three falls had a mean score of 5.7 (95% CI 4.8, 6.6). Finally, subjects who did not depend at all on another person to do daily activities had an index score of 2.1 (95% CI 1.9, 2.3) while those who depended on another person daily had mean scores of 4.9 (95% CI 4.3, 5.5). Each of these differences was highly statistically significant (p < 0.0001 for ordinal trend for each; Figure 2).

#### Independence of Association with Health Status

We performed a multiple linear regression in which the dependent variable was self-rated overall health, reported by subjects on a 0 to 100 scale (worst health imaginable to best health imaginable). Independent variables included age, sex, knee pain (measured with the WOMAC pain scale), mental health status, medical comorbidity, musculoskeletal functional limitations index score and an index of geriatric conditions including incontinence, fear of falling, muscle weakness and difficulty with vision, hearing or memory. The three most important independent predictors of self reported health status, as reflected in the t-statistics, partial correlations and p-values associated with each variable were the musculoskeletal functional limitations index, the medical comorbidity index and the mental health scale (Table 2). In a sensitivity analysis we added the Intermediate Activities of Daily Living Scale to the regression model. It had a strong relationship with self reported health status (p = 0.0001). With this ADL scale in the model, the musculoskeletal functional limitations index continued to have a significant association with self-reported health status (p = 0.0009).

**Table 2:** Variables associated with self-rated quality of life* in multivariable linear regression analyses

Independent variable	Parameter estimate	Standard error	T^	P-value	Partial Correlation
MSK functional limitations index	-1.31	0.22	-5.93	< 0.0001	0.23

WOMAC Pain	0.05	0.02	2.35	0.0190	0.09

Medical comorbidity	-2.54	0.49	-5.16	< 0.0001	0.20

Mental health	0.20	0.03	6.32	< 0.0001	0.24

Geriatric problems	-1.06	0.29	-3.64	0.0003	0.14

Age	-0.11	0.09	-1.15	0.25	0.05

Female gender	-1.23	1.05	-1.17	0.24	0.05

* self rated quality of life is dependent variable and ranges from 0 = worst health possible to 100 = best health imaginable

^T statistic has one degree of freedom for each variable

Among subjects with WOMAC Pain scores > 80 and WOMAC Functional Status scores > 70, the musculoskeletal functional limitations index ranged from 0 to 11. Half of these subjects had scores of 2 or greater and 22% had scores of 4 or greater.

## Discussion

We developed a six item self-report index of musculoskeletal functional limitation and administered it to a national sample of patients two years following total knee replacement. The index was developed by a multidisciplinary group of clinicians and methodologists ensuring content validity. The measure can be completed without assistance in 2–3 minutes. Our evaluation provides preliminary evidence of convergent validity at the item level and of convergent and discriminant validity at the index level.

Specifically, as hypothesized, the individual items have strong, monotonic associations with the Intermediate Activities of Daily Living Scale and the index score has moderately strong correlations (Spearman r = 0.44 to 0.64) with the WOMAC functional status scale, the Basic and Intermediate ADL Scores and a 0–100 rating scale of self-perceived overall health. The index distinguished subjects who use walking supports, experience falls, exercise regularly, and are dependent upon others for assistance from subjects who do not have these attributes. The index identifies musculoskeletal limitations even in patients with excellent WOMAC pain scores, indicating that it is not simply reflecting the results of TKR. The association between the musculoskeletal functional limitations index and self rated overall health persists after adjusting for the effects of age, pain, geriatric functional problems, mental health and medical comorbidity. Thus the musculoskeletal functional limitations index adds additional information not captured in these other more traditional measures.

Prior literature provides strong evidence for the effect of musculoskeletal problems on disability at the individual and population levels. The 1996 United Kingdom Survey of Disability documented that among the various conditions giving rise to disability, musculoskeletal disorders accounted by far for the greatest population-attributable fraction of disability (30%) [30]. Similarly, a population based survey in Finland documented that musculoskeletal disorders accounted for the largest losses in health related quality of life, followed by mental health disorders [14]. Similar findings have been reported from Australia, Italy, the United States and New Zealand, among other countries [19-21,31]. Despite these sobering findings of the pervasive effect of musculoskeletal problems on health outcomes, we are aware of no prior efforts to formally capture the number of functional limitations with a single score.

We performed our analyses in the subset of patients that completed all items on the index. In field settings, some patients will choose to leave certain items blank, for a variety of reasons. For example, in our study 16% of respondents left one or more items missing. The benefits and drawbacks of various options for addressing missing data likely vary across studies; hence investigators will need to make decisions best suited to their studies. We suggest in general that investigators consider the whole scale as missing if fewer than four of six items are completed, and that they calculate a mean for the completed items if four or five items are completed.

We have weighted each problem equally because the data in Figure 1 suggest similar impact on functional status. We rated the different levels of severity (no limitations, limited a little, limited a lot) as 0, 1 and 2. We recognize that alternative weighting schemes might influence the associations between index score and outcomes of interest. We did not explore alternative weighting schemes in this initial paper describing the index because there was no compelling clinical rationale. We also recognize that using the index as a single numerical variable in analyses may yield different findings from using is as a categorical variable. The issue of weighting and variable specification should be explored in future work on the index.

The strengths of this study include the large, national sample and the fact that subjects and research assistants were blinded to the hypotheses of the analyses. The sample of Medicare beneficiaries undergoing total knee replacement is apt because the subjects' age and advanced arthritis put them at risk for multiple musculoskeletal problems, yet the subjects are also medically stable enough to have been considered candidates for major surgery. The TKR population is useful for validation because these patients are at risk for other musculoskeletal problems, including osteoarthritis at other sites, as well as other comorbidities.

While this population offers distinct advantages, further work in patients with other chronic diseases across a broader age range would add to the generalizability of these analyses. In particular, further work should be done in populations that do not share in common prior exposure to a single orthopedic surgery, as this feature of our cohort may have influenced the pattern of musculoskeletal problems reported. For example, TKR patients are more likely to be female and obese than the general population. The study is also limited by lack of a physical examination to substantiate evidence of musculoskeletal limitation and by the predominance of lower limb symptoms in our sample. We were not permitted to contact patients multiple times over a short period and therefore were unable to document test-retest reliability in this sample. This is an important goal for future work with the index. We are reassured that self administered measures of comorbidity have been shown to be reproducible [3,7]. Our cross sectional design precluded using the index to predict future health status or utilization or to document responsiveness; these are important goal for future research using longitudinal designs. Finally, we were not able to exclude patients with subsequent TKRs and thus some of the limitations observed might arise from contralateral knee OA or TKR.

## Conclusion

We recommend further evaluation and validation in additional populations and settings, and in studies with a range of outcomes, including mortality and complications of management. For now, we conclude that the musculoskeletal functional limitations index has preliminary evidence of validity. We suggest that investigators use this measure to assess musculoskeletal functional limitations as a covariate and potential confounder in epidemiologic studies and trials involving patients with chronic disease.

## Competing interests

The authors declare that they have no competing interests.

## Authors' contributions

JNK led the design of the MSK functional limitations index, wrote the paper and contributed to devising the analytic policy. JAB provided methodological oversight and critically read the manuscript. EAW performed data management. EL led the statistical analysis and critically commented upon the paper.

## Pre-publication history

The pre-publication history for this paper can be accessed here:


